# A Low-Illumination Enhancement Method Based on Structural Layer and Detail Layer

**DOI:** 10.3390/e25081201

**Published:** 2023-08-12

**Authors:** Wei Ge, Le Zhang, Weida Zhan, Jiale Wang, Depeng Zhu, Yang Hong

**Affiliations:** National Demonstration Center for Experimental Electrical, School of Electronic and Information Engineering, Changchun University of Science and Technology, Changchun 130022, China

**Keywords:** low-illumination image enhancement, image decomposition, U-Net, Retinex-Net

## Abstract

Low-illumination image enhancement technology is a topic of interest in the field of image processing. However, while improving image brightness, it is difficult to effectively maintain the texture and details of the image, and the quality of the image cannot be guaranteed. In order to solve this problem, this paper proposed a low-illumination enhancement method based on structural and detail layers. Firstly, we designed an SRetinex-Net model. The network is mainly divided into two parts: a decomposition module and an enhancement module. Second, the decomposition module mainly adopts the SU-Net structure, which is an unsupervised network that decomposes the input image into a structural layer image and detail layer image. Afterward, the enhancement module mainly adopts the SDE-Net structure, which is divided into two branches: the SDE-S branch and the SDE-D branch. The SDE-S branch mainly enhances and adjusts the brightness of the structural layer image through Ehnet and Adnet to prevent insufficient or overexposed brightness enhancement in the image. The SDE-D branch is mainly denoised and enhanced with textural details through a denoising module. This network structure can greatly reduce computational costs. Moreover, we also improved the total variation optimization model as a mixed loss function and added structural metrics and textural metrics as variables on the basis of the original loss function, which can well separate the structure edge and texture edge. Numerous experiments have shown that our structure has a more significant impact on the brightness and detail preservation of image restoration.

## 1. Introduction

With the development of electronic devices, digital images have played an important role in our lives. They are widely used in fields such as traffic management, medicine [1], satellite remote sensing, and target recognition and tracking. However, the complexity of the shooting environment often leads to low-quality phenomena, such as low recognition, color distortion, and loss of details. Due to the low quality of images, subsequent computer vision tasks become difficult. Because image enhancement can improve the visibility and practicality of low-illumination images, it has important research value.

At present, image enhancement is mainly divided into traditional methods and deep-learning-based methods. Retinex theory, a model for brightness and color perception in human vision and a commonly used low-illumination image enhancement method, was proposed by Land [2] in the 1970s. Afterward, many scholars continued to build on this basis, from the single-scale Retinex (SSR) algorithm to the multi-scale Retinex (MSR) algorithm [3] and then to MSR with color recovery (MSRCR) [4]. However, both SSR and MSR generally exhibit color distortion. Compared with other algorithms, the MSRCR algorithm has a better color restoration ability, but it has high computational complexity and many adjustable parameters that are difficult to adaptively select. In addition, on the basis of Retinex theory, some simple and efficient image enhancement methods based on the Retinex model have been proposed for low-illumination image enhancement [5], such as LIME [6], RobustRetinex [7], and JED [8]. Reference [9] also proposed a convex variational model, which could effectively decompose the gradient field of an image into prominent edges and a relatively smoother illumination field through first- and second-order total change regularization. In summary, although these traditional methods can effectively enhance image brightness and preserve high-frequency information such as edges and corners, they cannot effectively avoid problems such as uneven contrast and color distortion.

In recent years, enhancement methods based on deep learning have gradually developed. The first network based on deep learning to solve low-light image enhancement was LLNet [10]. Paired images, including low-light images and normal images, were input into the network model and trained through automatic encoders to learn the basic signal features in low-light images and adaptively improve the brightness and denoising ability. This method loses details during image reconstruction, resulting in a slightly blurry enhancement of the final image. Afterward, the Retinex-Net network model was proposed by combining Retinex theory with divine-level convolutional networks [11]. Firstly, an image was decomposed into illumination maps and reflection maps through the decomposition network. Secondly, the illumination image was enhanced through an enhancement network, and finally, the enhanced illumination image was multiplied by the decomposed reflection image to obtain the final enhanced image. After Retinex-Net, an author proposed a method to improve the quality of low-light images by analyzing the histogram of the images and utilizing deep learning techniques. For example, the MBLLEN [12] algorithm is a low-light image enhancement algorithm based on a multi-branch network. This algorithm extracts rich image features from different levels, enhances images through multiple sub-networks, and finally generates output images through multi-branch fusion. Another algorithm is the EnlightenGAN [13] algorithm, which improves the quality of low-light images through local discriminators and attention modules. This algorithm has shown good enhancement effects in real scenes, but there are still some shadow areas in some images. Kind [14] used a trainable denoising module for reflectivity recovery. In addition, a learnable mapping function was designed in the lighting adjustment module, where images could be flexibly restored at user-specific lighting levels. Sci [15] adopted a new self-adjustment lighting framework and established a cascaded lighting learning process with weight sharing to achieve fast and flexible image enhancement. These methods all have good enhancement performance. In real-world scenes, unclear details and inappropriate exposure are common. However, the existing methods fail to solve the above problems.

The proposed method draws inspiration from the Retinex theory [16]. The Retinex model can divide an image into two parts: the incident component and the irradiation component. Specifically, the irradiation component reflects the distribution of light in the shooting environment. The reflection component represents the essential properties of an image. In this paper, the image is decomposed into two parts: a structural layer and a detail layer. The structural layer mainly refers to the main contour or global geometric structure information of the image, and the clear boundaries and connected regions are the main reasons for light decay. The detail layer refers to the image containing small scales and details, which are usually periodic and oscillatory. Based on the above ideas, a low-illumination image enhancement method based on a structural layer and a detail layer is proposed. The main contributions of this article include:

(1) This proposed SRetinex-Net model is mainly divided into two parts: a decomposition module and an enhancement module. The decomposition module mainly adopts the SU-Net structure, which decomposes the input image into a structural layer image and a detail layer image. The enhancement module mainly adopts the SDE-Net structure, which is divided into two branches: the SDE-S branch and the SDE-D branch. The SDE-S branch mainly enhances the brightness of the structural layer, while the SDE-D branch enhances the textural detail of the detail layer.
(2) The SU-Net structure is an unsupervised network, which mainly extracts and merges the structural features of input images through a sampling layer and skip connection. A brightness calibration module was added to the SDE-S branch. After the brightness enhancement of the structural layer image through the Ehnet module, the feature extraction and reconstruction of the enhanced image should be completed through the Adnet module to adjust the image brightness, making the image brightness more balanced and accurate. The SDE-D branch is mainly denoised and enhanced with detailed textures through a denoising module. This network structure greatly improves computational efficiency.
(3) The total variation optimization model was improved as a mixed loss function, and the structure component and texture component were added as variables on the basis of the original loss function, which can make the edge and texture better separated so that the edge of the structural layer image is clear and the details of the detail layer image are more abundant.
(4) Compared with previous methods, the structural layer image structure obtained by decomposing the image is more complete in preservation, and the detail layer image contains more abundant details. In image enhancement, our method does not refer to normal light images. We can adaptively adjust image brightness to better match human visual effects and have conducted extensive experimental comparisons to demonstrate the superiority of our method. Compared with all other methods, we can self-calibrate image brightness, enhance image contrast, and improve image details and visibility.

## 2. Methods

The low-illumination image enhancement method based on convolutional neural networks makes it difficult to generate complete details during image reconstruction, which can easily lead to slightly blurry enhancement results. To solve this problem, this paper proposes a low-illuminance enhancement method based on decomposing the image into a structural layer and a detail layer. First, the color space of the source image is transformed from RGB to HSV. Then, the V image component is decomposed into a structural layer and a detail layer. Furthermore, the structural layer image’s brightness is enhanced through structural branching, while the detail layer image’s textural details are enhanced through detail branching. Finally, the enhanced structural layer and detail layer are multiplied to obtain the enhanced V-component image. The enhanced V-component image is combined with the H- and S-component images and transformed into a color space to obtain the final low-illumination-enhanced image.

### 2.1. Framework of the Proposed Method

Firstly, the color space of the source image is transformed from RGB to HSV. Secondly, the source image is decomposed into the H-, S-, and V-channel components, which can be referred to as I_h_, I_s_, and I_V_, respectively. Finally, the H and S image channels remain unchanged, and the V image channel is extracted as the input to the network. Afterward, the input image I_V_ can be decomposed into the structural layer I_Vs_ and detail layer I_Vk_ via the decomposition module, which is the input of the enhancement module. Next, the structural layer I_Vs_ is fed into the SDE-S branch to enhance the brightness. The detail layer I_Vk_ is fed into the SDE-D branch to enhance the details. Then, the brightness enhancement image I_Vs_′ and the detail enhancement image I_Vk_′, which are the outputs of the two branches, are multiplied to obtain the enhanced image I_V_′ of the V-channel component. Finally, the final enhanced image I′ is obtained by fusing the components of the I_h_, I_s_, and I_V_′ channels and converting it from the HSV space to the RGB space.

**Figure 1:**
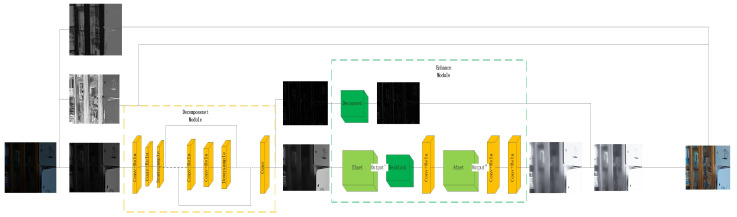
Framework of the proposed method.

As shown in Figure 1, the proposed method can enhance and maintain the detail information of an image while enhancing the brightness and contrast of the image, ensuring the visual quality of the enhanced image.

### 2.2. Structure of the Network

#### 2.2.1. Decomposition Module

Compared with the Retinex-based method, the decomposition model decomposes the input image into structural layer images I_Vs_ and detail layer images I_Vk_, rather than illumination and reflection images. Therefore, we do not need to label images with normal brightness to constrain the network training.

The original U-net structure [17] consumes considerable training time and has the problem of repeated training. Because there are no labels for the structural layers and detail layers of the trained images, we need to retrain each image during training. In order to satisfy this condition, a SU-Net structure is proposed, which mainly uses multiple convolution layers and a nonlinear activation function connection, including an upper sampling layer, lower sampling layer, and skip connection. Because we input a single-channel image, the first layer of convolution has 1 input channel and 64 output channels, the last layer of convolution has 64 input channels and 1 output channel, and the remaining convolution has 64 input and output channels. The entire network completes the feature extraction and reconstruction of images.

#### 2.2.2. Enhancement Module

**Figure 2:**
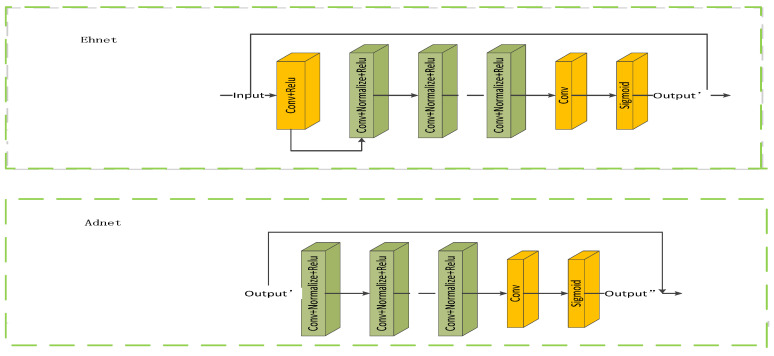
Brightness enhancement network and brightness adjustment network.

The enhancement module adopts an SDE-Net structure. The network is divided into two branches: the SDE-S branch and the SDE-D branch. The SDE-S branch mainly enhances the brightness of the structural layer I_vs_ to obtain the enhanced structural layer image I_Vs_′, while the SDE-D branch enhances the textural details of the detail layer I_vk_ to obtain the enhanced detail layer image I_Vk_′. Ehnet in the SDE-S branch is mainly composed of multiple convolution layers and an activation function, and the size of the convolution kernel is 3 × 3. The input channel number of the first convolutional layer is 1, and the output channel number of the last convolutional layer is 1. It mainly performs feature extraction and reconstruction on the input image to enhance image brightness. Adnet is a brightness adjustment network that receives a preliminary brightness-enhanced images output via Ehnet, performs feature extraction and reconstruction on the input image to adjust the brightness of the image, prevents image brightness overexposure or insufficient brightness, and makes the brightness of the image more balanced and accurate. Adnet mainly consists of blocks composed of multiple convolutional layers, each with two 3 × 3, a reduction layer, and an activation function. The number of input channels of the first convolution layer is 1, and the number of output channels of the last convolution layer is 1. The SDE-D branch enhances the texture details of the detail layer I_vk_ through a noise reduction module. This approach can greatly improve computational efficiency, as shown in Figure 2.

### 2.3. Loss Function

#### 2.3.1. Fully Variational Loss Function

The image can be divided into different image layers based on various methods. For example, the image can be divided into high-frequency and low-frequency signals based on frequency domain methods, and the original image can be decomposed into illumination images and reflection images based on the Retinex algorithm. The image can be decomposed into structural layers and detail layers. The structural layer mainly refers to the main contour or global geometric structural information of the image, with clear boundaries and connected regions. The detail layer refers to a layer that contains small scales and details, which are typically periodic and oscillatory. There are many methods for image decomposition, and image filters can be used for filtering, such as the rolling filter algorithm [18]. A Gaussian filter is used to remove texture, while bilateral filters are used to restore edges, which also causes ringing and artifacts around the edges. Image decomposition can also be achieved using methods such as the TV (total variation) full variation model [19] and the relative total variation (RTV) model [20].

Herein, we use the total variation model as the basis for the optimization framework, and the common total variation objective formula is as follows:

(1) $$S=arg\underset{s}{\min}\sum_{i}\left({∥u(i)-I(i)∥}_{m}+∥\beta \nabla u(i){∥}_{n}\right)$$

We represent the intensity of the input image, its structural layer components, and its detail layer components as I, S, and K, respectively. Our goal is to obtain unknown structural layer images S and detail layer images K from known input images I. Because I = S + K, we only need to estimate one of S and K. In the variational framework, the structural component S is generally obtained by changing the feature metric of the fully variational model, such as in references [21,22,23], where i represents the pixel intensity at the point and is a balance coefficient, and the subscripts m and n represent the function space of the two terms. In Formula (1), the first term is the fidelity term, which mainly makes the structural layer S infinitely close to the input image I. The second term is the regularization term, which is mainly used to remove the edges in the structural layer diagram.

In order to better decompose the image structural layer and detail layer, considering the anisotropy of image gradients, structural metrics [24] and textural metrics [24] are used to optimize the total variational function.

The structural measurement formula is as follows:

(2) $$G_{s}\left(i\right)=A_{J}\left(i\right)\frac{∥\nabla f\left(i\right){∥}_{1}}{R},$$

where $A_{J}(i)$ represents the degree of anisotropy in the local area of point i, j represents the positive definite matrix, and a larger value of A indicates that the degree of anisotropy and structural strength at that point is stronger. On the contrary, a smaller value of A indicates a smaller degree of anisotropy and stronger texture details at that point. $∥\nabla f(i){∥}_{1}$ represents the L1 norm of the gradient of the image at point i, and R represents the maximum value.

The texture measurement formula is as follows:

(3) $$G_{t}(i)=\frac{1}{\left\{C(i)\right\}}*\sum_{j\in \left\{C(i)\right\}}\cos\theta_{ij}*e^{\left(-\Phi (h_{j},h_{i})\right)},$$

where $\left\{C(i)\right\}$ represents the set of domain pixels of point i, j represents the domain position of pixel point i, and $-\Phi (h_{j},h_{i})$ represents the cross-entropy. $\cos\theta_{ij}$ represents the edge direction positions of pixel points i and j. When i and j are on the same edge, the included angle is 90 degrees. Conversely, when the included angle is 0, it has no effect on the texture measurement. The range of values for $G_{t}$ is [0, 1].

Therefore, the objective function we utilized is as follows:

(4) $$S=arg\underset{s}{\min}\sum_{i}\left({∥u(i)-I(i)∥}_{1}^{2}+D(i)∥\nabla u(i){∥}_{1}\right),$$

(5) $$D\left(i\right)=[\beta_{s}\left(1-G_{s}\left(i\right)\right)+\beta_{t}G_{t}\left(i\right)],$$

where S represents the decomposed structural layer, I represents the original input image, ${∥u(i)-I(i)∥}_{1}^{2}$ refers to the difference between the input image and the output structural layer image, $D\left(i\right)$ refers to the i-point structural and textural metrics, ∇u(i) is the gradient of the structural components at the i-point, and ${∥∥}_{1}^{2}$ is the Lp norm. $G_{s}$ is a structural metric responsible for filtering out structural edges. When the structural metric value of point i is large, that is, $G_{s}$ approaches 1, and 1- $G_{s}$ approaches 0, the gradient regularization term also approaches 0. Therefore, the structural edges of point i can be retained. In this case, the influence of the second term should be reduced; on the contrary, when the texture measurement value of point i is large, that is, $G_{t}$ tends to 1, and the gradient regularization term is large, the texture edge at point i can be separated from the structure component. At this point, the second main function is to effectively remove textural edges. $\beta_{s}$ and $\beta_{t}$ are the equilibrium coefficients of $G_{s}$ and $G_{t}$.

We defined the loss function as the objective function (4) and (5) and trained the neural network. The fully variational loss function Formula (6) is as follows:

(6) $$loss=\sum_{i}\left({∥u(i)-I(i)∥}_{1}^{2}+D(i)∥\nabla u(i){∥}_{1}\right),$$

Because we do not have the label of the structural layer image, the unknown parameters in the loss function are adjusted with the input image.

**Figure 3:**
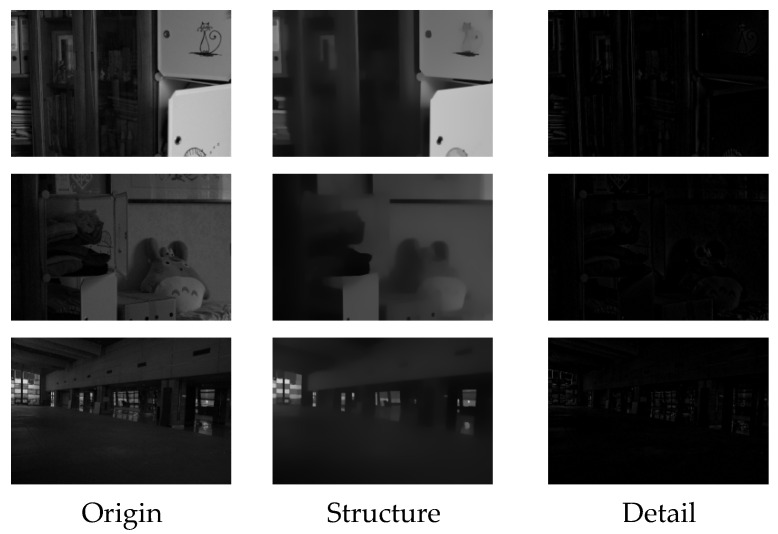
Structural layer images and detail layer images.

The structural layer images and detail layer images obtained using the decomposition module are shown in Figure 3.

#### 2.3.2. Unsupervised Loss Function

This enhancement method can avoid the uncertainty of paired data sets, and we used the unsupervised loss function [15] to achieve this purpose.

(7) $$L_{un}=\gamma_{1}L_{f}+\gamma_{2}L_{s},$$

where $L_{f}$ and $L_{s}$ represent the fidelity loss and smoothing loss. $\gamma_{1}$ and $\gamma_{2}$ are two positive equilibrium parameters.

The fidelity loss function ensures that the estimated illumination is consistent with the pixel level between the inputs of each stage. The specific formula is as follows:

(8) $$L_{f}=\sum_{t=1}^{T}{∥x^{t}-(y+s^{t-1})∥}^{2},$$

where T represents the total number of stages. In fact, the fidelity loss function uses the redefined input to constrain the output lighting rather than the live scene or normal low-light input photographed artificially. X represents the generated illumination estimation, y represents the low-illumination image to be processed, and s represents the adjustment parameters.

The formula of the illumination smoothing loss function is as follows:

(9) $$L_{s}=\sum_{i=1}^{N}\underset{j\in N(i)}{\sum }w_{i,j}\left|x_{i}^{t}-x_{j}^{t}\right|$$

where N represents the total number of pixels. I is the i-th pixel. N (i) represents point 5 of i × adjacent pixels in the range of 5. X represents the generated illumination estimation image, and $W_{i,j}$ represents the weight between pixels i and j, which is used to measure the similarity between pixels i and j.

## 3. Experimental Results and Analysis

To verify the effectiveness of the proposed method, our low-illumination image enhancement method based on structural and detail layers was compared with existing classic algorithms as a comparative experiment, and validation analysis was conducted based on two aspects: subjective visual effects and objective evaluation indicators. In order to verify the generalization of the network, this article used the publicly available LOL dataset and MEF dataset as training datasets. The LOL dataset contains 485 pairs of low-light/normal-light training images and 15 low-light test images. The MEF dataset contains 84 low-light test images.

In the pre-training process of the decomposition module, data preprocessing is performed first. The color space of the source image is transformed from RGB to HSV, and the V-component image is extracted. Then, structural and textural metrics are calculated separately for each V-component image, and the experimental results are saved. Afterward, the network is used for pre-training. The structural metric and detail metric of each training image remain unchanged, and the balance coefficient in the loss function formula is 3.0. A total of 30 iterations are conducted in the pre-training stage. At this time, the network is considered to converge, and the pre-training is complete.

There are two parts to the enhancement module: pre-training and fine-tuning. The structural layers obtained from the decomposition module are pre-trained with 1000 iterations and a learning rate of 0.0003. After approximately 396 iterations, the network converges, and the pre-training ends.

### 3.1. Subjective Evaluation

**Figure 4:**
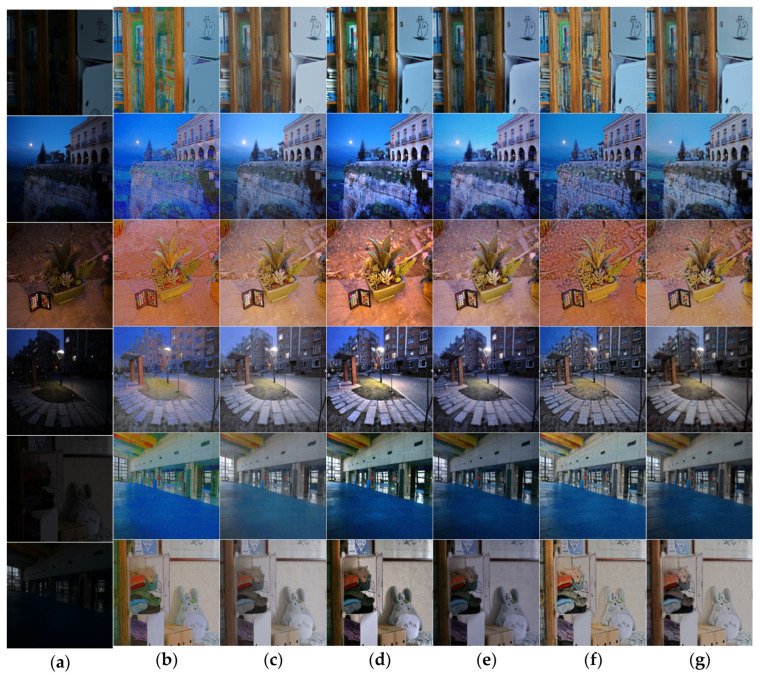
Comparison of low-illumination image enhancement effects of different algorithms. (**a**) Origin images, (**b**) RetinexNet results, (**c**) UretinexNet results, (**d**) LIME results, (**e**) Zero-Dce++ results, (**f**) KinD++ results, and (**g**) our results.

In terms of subjective visual effects, as shown in Figure 4, six groups of images are selected, including indoor scenes and natural landscapes. From left to right, there are enhancement images of low-illumination images, the Retinex-Net algorithm, URetinex-Net algorithm, LIME algorithm, Zero DCE++ algorithm, Kind++ algorithm, and the algorithm presented in this article.

**Figure 5:**
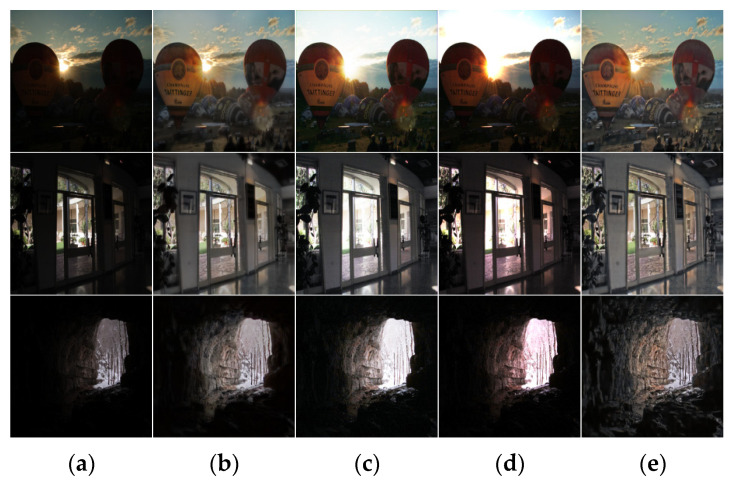
Comparison of low-illumination image enhancement effects of different algorithms. (**a**) Origin images, (**b**) Kind results, (**c**) Sci results, (**d**) RUAS results, and (**e**) our results.

As shown in Figure 4 and Figure 5, it can be seen that the enhanced images of the Retinex-Net algorithm exhibit significant color distortion, with some images exhibiting a noticeable ink sensation. The URetinex-Net algorithm [25] enhances the overall image and has certain defects in image color retention. Many objects tend to have obvious fading phenomena. The LIME algorithm has an excessive enhancement effect on local regions. Zero DCE++ [26] has a poor noise suppression effect and is prone to detail loss. The KinD++ algorithm [27] significantly improves the brightness, but the brightness of the enhanced image cannot maintain the same brightness distribution characteristics as the original image, and there is obvious color distortion. The proposed method in this article has a more reliable enhancement effect, which can work well under different types of lighting conditions, effectively avoiding situations where the overall vision is too high or the enhancement is insufficient. The final enhancement effect is also more natural and realistic.

**Figure 6:**
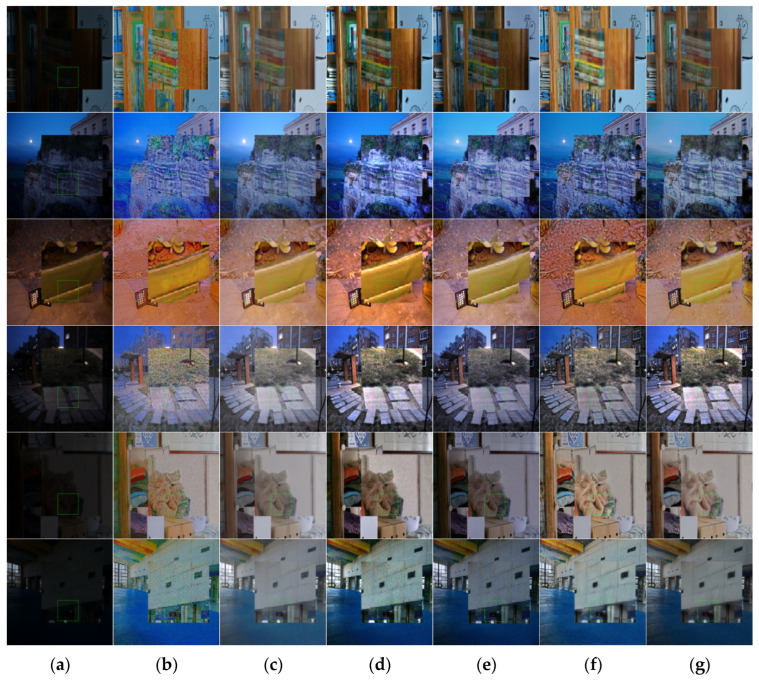
Comparison of local enlargement details of low-illuminance images using different algorithms. (**a**) Origin images, (**b**) RetinexNet results, (**c**) UretinexNet results, (**d**) LIME results, (**e**) Zero-Dce++ results, (**f**) KinD++ results, and (**g**) our results.

**Figure 7:**
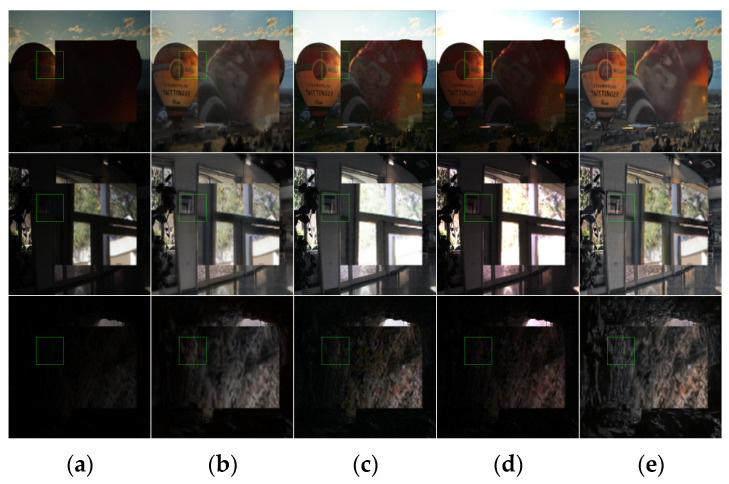
Comparison of local enlargement details of low-illuminance images using different algorithms. (**a**) Origin images, (**b**) Kind results, (**c**) Sci results, (**d**) RUAS results, and (**e**) our results.

The details of Figure 4 and Figure 5 are enlarged in Figure 6 and Figure 7. In the figures, it can be seen that the results obtained with the Retinex-Net algorithm show color distortion and excessive detail enhancement in some areas, such as the bookcase area and cliff area in the image, which are biased toward ink and have artifacts. The URetinex-Net algorithm shows a significant color bias toward white in the enlarged area of the flowerpot and clothing. The LIME algorithm clearly shows the presence of a large amount of noise in the enlarged area of streetlights and swimming pools. The Zero-DCE++ algorithm shows that the contrast enhancement is not sufficient in the enlarged area, resulting in a dim overall color sense in the image and an obvious problem of detail loss. The KinD++ algorithm has the problem of overexposure in the magnified area of the natural landscape. The magnified area of the indoor scene recovers the color distortion, and the brightness recovery is unstable. The proposed method in this article utilizes the advantages of the HSV color space compared with the other methods. While maintaining the structure, it preserves most of the original information of the images and enriches the details of the objects, avoiding color distortion to the greatest extent. At the same time, it can effectively suppress the generation of noise and avoid the presence of artifacts.

### 3.2. Objective Evaluation Indicators

In order to better evaluate image quality, this article used the natural image quality evaluator (NIQE) [28], structural similarity index (SSIM), peak signal-to-noise ratio (PSNR), and learned perceptual image patch similarity [29] (LPIPS) to evaluate the resulting images. As an evaluation indicator, the higher the values of the SSIM and PSNR, the better the image quality we will obtain; on the contrary, smaller NIQE and LPIPS values indicate better image quality. BIQI is an image evaluation index without reference images, with values ranging from zero to one. The closer the value is to one, the better the image quality. The EMEE evaluation indicator is used to measure image edge information, especially for images with clear edges. The EMEE value is small, and vice versa. SDME is an image evaluation indicator used to measure the degree of edge change in images. A larger value indicates a more significant edge change in the image. BRISQUE is a five-reference image quality evaluation indicator, with values typically ranging from 50 to 100. The larger the value, the better the image quality. The AME evaluation indicator is suitable for measuring the quality of image edges, and the value is usually positive. Images with clear edges have a higher AME value, while the opposite is true. Visibility is an indicator of image visibility, with larger values indicating clearer targets or details in the image, and vice versa.

The enhanced results of the test datasets are shown in Table 1. **↑** the larger the value, the better the enhancement effect. On the contrary, **↓** the smaller the numerical value, the better the enhancement effect. As shown in the table, our method is better than the other methods in the NIQE, SSIM, PSNR, BIQI, EMEE, BRISQUE, AME, and visibility metrics, except that it performs slightly worse than URetinexNet and LIME in LPIPS and SDME. In summary, we have achieved an effective solution to the existing problems, and the results are excellent.

### 3.3. Ablation Experiment

The loss function variable in the decomposition module, the brightness enhancement module, and the adjustment module in the enhancement module of the network model in this paper were ablated. The specific experimental results are shown in the following figure.

**Figure 8:**
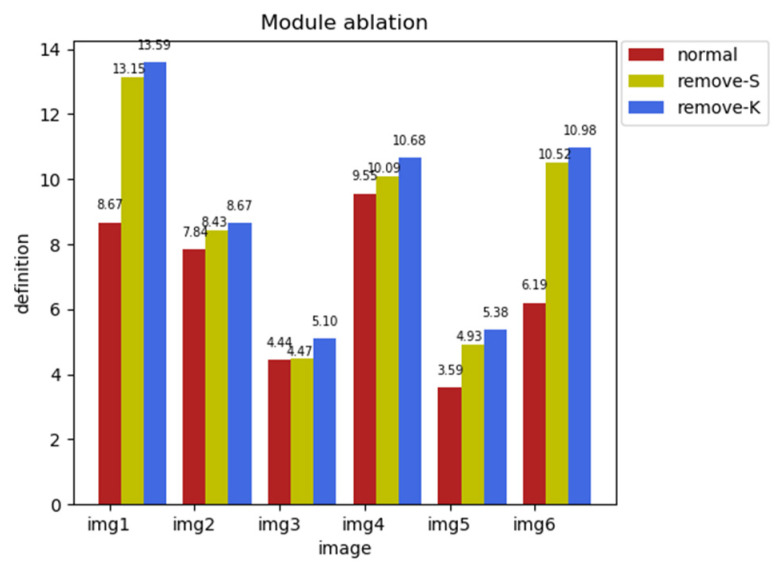
Ablation experiment for loss function variable.

As shown in Figure 8, in the ablation experiment for the loss function variables, which was mainly to verify the importance of structural metrics and textural metrics for the generation of structural layers, clarity was used as the key to measure the effects of the variables. Clarity refers to the details and boundaries in an image, and higher values represent more detailed information contained in the image. For the structural layer, the less detailed information we have, the better our final enhancement effect. Therefore, we need to choose variables with smaller clarity values. The red color in the histogram indicates that the loss function variables include both structural metrics and textural metrics; yellow indicates that the loss function variable only contains textural metrics; blue indicates that the loss function variable contains only structural measures. As can be seen in Figure 8, only the loss function containing structural metrics and textural metrics obtains the best structural layer effect.

**Figure 9:**
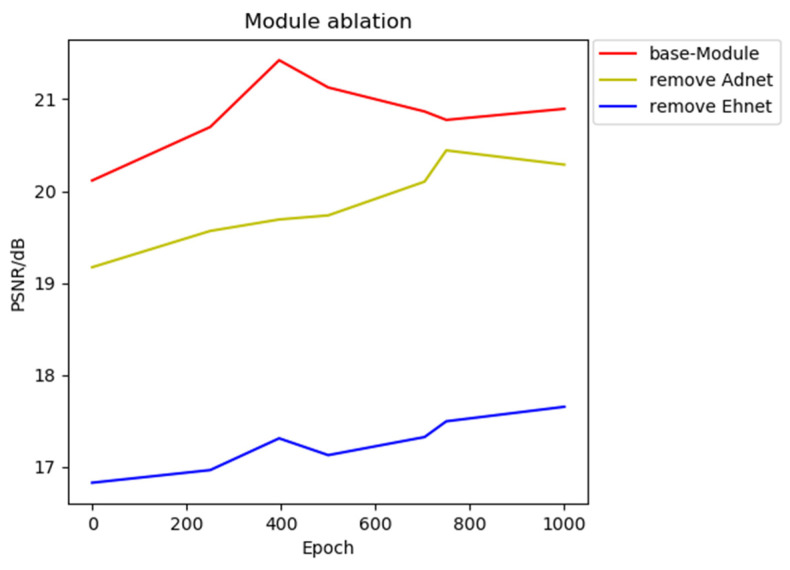
Ablation experiment based on enhancement module.

Figure 9 shows the module ablation experiment for the second part of the network structure enhancement module, mainly comparing the basic module, the removed adjustment module, and the removed enhancement module. The peak signal-to-noise ratio is used as the key to measuring the experimental structure. The higher the peak signal-to-noise ratio, the stronger the enhancement effect. Therefore, we need to choose a module with a higher peak signal-to-noise ratio value. As shown in Figure 6, red represents the basic module, yellow represents the removal of the brightness adjustment module, and blue represents the removal of the enhancement module. It can be clearly seen that only the enhancement module and brightness adjustment module coexist, and the network has the best enhancement effect.

## 4. Conclusions

This paper proposed a low-illumination enhancement method based on structural and detail layers. Firstly, we designed an SRetinnex-Net model. The network is mainly divided into two parts: a decomposition module and an enhancement module. Second, the decomposition module mainly adopts the SU-Net structure, and the network decomposes the input image into a structural layer image and detail layer image. Afterward, the enhancement module mainly adopts the SDE-Net structure, which is divided into two branches: the SDE-S branch and the SDE-D branch. The SDE-S branch mainly enhances and adjusts the brightness of the structural layer image through the Ehnet module and the Adnet module, to prevent insufficient or overexposed brightness enhancement in the image. The SDE-D branch is mainly denoised and enhanced with textural details through a denoising module. This network structure can greatly reduce computational costs. Moreover, we also improved the total variation optimization model as a mixed loss function and added structural metrics and textural metrics as variables on the basis of the original loss function, which can well separate the structure edge and texture edge. Numerous experiments have shown that the algorithm proposed in this paper outperforms Retinex-Net, SIRE, LIME, Zero-DCE++, Kind++, RUAS, and other algorithms in evaluation metrics such as the SSIM, PSNR, and NIQE. The algorithm proposed in this article not only improves the brightness of low-illumination images but also has significant advantages in enhancing textural details and color restoration. In the future, the decomposition and enhancement of the entire network play an important role in enhancing low-illumination images, and optimizing the network structure is also a focus of our future research direction. And for low-illumination images without a control group, how to ensure image brightness enhancement without losing image details is a major challenge for us to continue studying low-illumination image enhancement.

## Figures and Tables

**Table 1:** Objective evaluation indicators of different algorithms.

Comparison Algorithm	NIQE ↓	SSIM ↑	PSNR ↑	LPIPS ↓	BIQI ↑	EMEE ↑	SDME ↑	BRISQUE ↓	AME ↑	Visibility ↑
Retinex-Net	7.1888	0.6449	13.7448	2.3146	0.4075	9.1803	89.1120	93.0386	78.9014	1.4980
URetinex-Net	4.7599	0.8238	21.3282	1.3234	0.2692	8.8664	72.2450	94.4427	43.9180	1.3153
SIRE	6.2109	0.4937	10.9447	1.8563	0.3428	8.4146	52.3258	93.3717	37.7913	1.5000
LIME	6.4282	0.7410	16.2744	2.0601	0.3436	7.9899	114.8789	94.8650	83.0246	1.3913
Zero-DCE++	4.3693	0.5479	14.3098	1.8905	0.3604	7.8689	69.8208	94.3531	52.4144	1.4879
KinD++	4.8106	0.7962	15.2666	1.4899	0.3652	8.5482	97.2805	93.3560	73.7956	1.4440
SNR-Aware [30]	5.7982	0.7834	17.3118	1.6384	0.3073	8.6534	68.6665	96.2248	58.0088	1.2607
RUAS [31]	6.2769	0.6075	12.9109	1.9274	0.2815	9.8992	65.1625	95.7833	50.9025	1.4222
OURS	4.3195	0.8321	21.4243	1.3882	0.4394	10.9775	110.7982	92.1687	83.1254	1.5169

## Data Availability

The data presented in this study are available on request from the corresponding author. The data are not publicly available due to the privacy of the institute.
